# WC-1 and the Proximal GATA Sequence Mediate a Cis-/Trans-Acting Repressive Regulation of Light-Dependent Gene Transcription in the Dark

**DOI:** 10.3390/ijms20122854

**Published:** 2019-06-12

**Authors:** Andrea Brenna, Claudio Talora

**Affiliations:** 1 Department of Biology, Biochemistry, University of Fribourg, 1700 Fribourg, Switzerland; 2 Pasteur Cenci Bolognetti Foundation c/o Department of Biology and Biotechnology “Charles Darwin”, Sapienza University, 00185 Rome, Italy; 3 Department of Molecular Medicine, Sapienza University of Rome Viale Regina Elena 291, 00161 Roma, Italy; Claudio.Talora@uniroma1.it

**Keywords:** WC-1, zinc finger, GATA, light responses

## Abstract

**Simple Summary:**

We observed that both the proximal GATA sequence in light-responsive elements (LREs) of the *albino-3* promoter and the Zinc Finger Domain of WC-1 are involved in the dark-related repressive control mechanism of light-regulated genes.

**Abstract:**

Light influences a wide range of physiological processes from prokaryotes to mammals. *Neurospora crassa* represents an important model system used for studying this signal pathway. At molecular levels, the WHITE COLLAR Complex (WCC), a heterodimer formed by WC-1 (the blue light photo-sensor) and WC-2 (the transcriptional activator), is the critical positive regulator of light-dependent gene expression. GATN (N indicates any other nucleotide) repeats are consensus sequences within the promoters of light-dependent genes recognized by the WCC. The distal GATN is also known as C-box since it is involved in the circadian clock. However, we know very little about the role of the proximal GATN, and the molecular mechanism that controls the transcription of light-induced genes during the dark/light transition it is still unclear. Here we showed a first indication that mutagenesis of the proximal GATA sequence within the target promoter of the *albino-3* gene or deletion of the WC-1 zinc finger domain led to a rise in expression of light-dependent genes already in the dark, effectively decoupling light stimuli and transcriptional activation. This is the first observation of *cis*-/*trans*-acting repressive machinery, which is not consistent with the light-dependent regulatory mechanism observed in the eukaryotic world so far.

## 1. Introduction

Because of the ability to perceive light, organisms can sense the change of time and, therefore, anticipate their physiological needs [1,2,3]. During their evolution, fungi developed sensory molecules able to detect the light signal and transduce it to regulate cell division and metabolism [4]. *Neurospora crassa* (*N. crassa*) can perceive only the near-UV/blue light spectrum [5,6]. Blue light influences many physiological processes in *Neurospora* including entrainment and resetting of the circadian clock, the induction of sexual conidiospores, or the biosynthesis of carotenoids [7,8,9]. Light-induced responses are processes generating hierarchical cascades that involve regulation of gene clusters grouped in two large classes. These genes are defined as early light responders (ELRs) and late light responders (LLR). ELRs show an average peak in mRNA expression at approximately 15 to 30 min after light stimulation. Genes regulated in this way are the main circadian clock component *frequency* (*frq*), genes involved in carotenoid biosynthesis like *albino-1* to *albino-3* (*al-1–al-3*), or photoadaptation genes like *vivid* (*vid*) [5,10,11,12,13,14]. In LLRs, mRNA expression peaks between 60 and 90 min after light stimulation [15]. It has been estimated that 5.6% out of 5600 detected genes on a whole-genome microarray can respond to light [10]. Extensive molecular and phenotypical screening led to the identification of only two genes, called *wc-1* and *wc-2*, whose mutations abolished all light responses. These genes encode for two proteins called WHITE COLLAR-1 (WC-1) and WHITE COLLAR-2 (WC-2) [16,17]. Both WC-1 and WC-2 contain Pest Arnt Sim (PAS) domains that serve as versatile sensors and interaction modules in signal transduction, and they are necessary for their heterodimerization [18]. The preassembled WHITE COLLAR Complex (WCC) translocates into the nucleus, where it is recruited onto the promoter sequences of target genes in the dark but, only after a light pulse, the WCC starts to activate their expression [19]. Additionally, WC-1 acts as a blue light Flavin Adenine Dinucleotide (FAD)-binding photoreceptor, which can transduce the blue light signal in *N. crassa* [20]. This is possible because of one of its modified PAS domains, the Light Oxygen Voltage (LOV) domain. About 20% of all annotated transcriptional factors in *Neurospora* are regulated during the early light response by the WCC [21]. In addition, light-inducible promoters show transient changes in the acetylation level of lysine 14, following the typical oscillation of light-dependent processes [22]. This light-dependent regulation of the chromatin is mediated by the physical interaction between WC-1 and *Neurospora* Gcn Five-1 (NGF-1), homologous to *Saccharomyces cerevisiae* Histone Acetyl Transferase HAT Gcn5p in a way that resembles the nuclear receptor/coactivator interaction [23,24]. WC-1 and WC-2 also contain two zinc finger transcription factor plant-like domains [16,17]. As transcription factors, both WC-1 and WC-2 bind specific promoter regions called light-responsive elements (LREs). GATN (Guanine, Adenine, Thymine, N is any other nucleotide) consensus elements within promoter sequences characterize these regions. LREs are classified as either Distal-LRE (D-LRE) or Proximal-LRE (P-LRE), taking into consideration the distance from the transcriptional start site [12,25,26]. The first LRE is also called C-box, and it is required for both circadian activation and light regulation [27,28]. Although the role of WC-2 as a light-related transcription factor is clear, the position of WC-1 in this molecular model remains controversial [29,30]. WC-1 is capable of binding to the *al-3* promoter fragment in vitro in band shift experiments; however, it is unclear how it could influence light-dependent gene transcription [16,17,18]. For instance, a *Neurospora wc-1* strain lacking the zinc finger domain has an impaired circadian rhythm, but it is still capable of gene activation by light [11,30].

Moreover, WC-1 overexpression is insufficient to activate most light-inducible genes even when it is enough to activate circadian clock-associated genes [31]. Even though we have a general picture of light responses in *Neurospora*, some aspects still appear unclear, and essential information is missing. For instance, it is still not clear what the role of the two GATA sequences is within the promoters of light-dependent genes such as *al-3*. So far, we only know that the distal one is also involved in circadian phenomena. To get new insights about the molecular model, we performed *cis* (mutagenizing GATA sequences) and *trans* (analysis of strains with a deletion of the WC-1 zinc finger domain) experiments. Our data give new evidence about an unclarified control mechanism of the dark/light shift mediated by WC-1 and the proximal GATA consensus sequence. These data suggest a novel role of WC-1, which does not work as an activator (as assumed but never proved so far) but as a dark-dependent transcriptional repressor. 

## 2. Results

### 2.1. WC-1 is Essential for the Proper Assembly of White Collar Complex (WCC) on Light-Regulated Promoters (LRPs)

**Figure 1:**
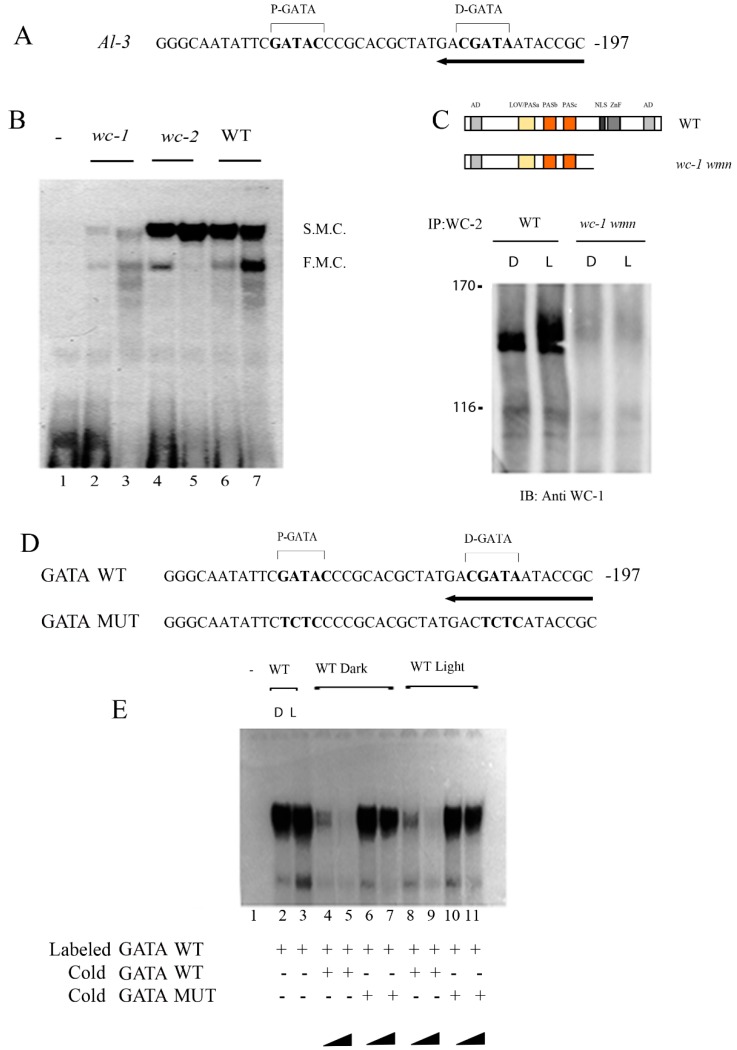
Dark/Light binding of WHITE COLLAR Complex (WCC) to the light-responsive elements (LREs) of *al-3*. (**A**) The DNA sequence of LRE in the promoter of *al-3* used as a radioactive probe for the Electrophoresis Mobility Shift Assay (EMSA) assay. The sequence was based on previously published data [26]. The arrow indicates the direction towards the transcriptional start site. (**B**) EMSA assay was performed incubating a double-stranded oligonucleotide probe containing the LREs of *al-3* promoter and a nuclear extract from *Neurospora crassa*. Lane 1 does not contain extract. In lanes 2–3 the probe was incubated with the nuclear extract obtained from *wc-1* null mutants grown either in the dark (lane 2) or light-treated for 20 min (lane 3). Same conditions were used in lanes 4–5 (*wc-2* mutant, 234 w) and 6–7 (wt). (**C**) Interaction of WC proteins is dependent on WC-1 C-Term region. WC-2 protein was immunoprecipitated with an anti-WC-2 antiserum from wt or *wmn* [23] (grown in the dark or 20 min after the light pulse. The immunoprecipitated complexes were resuspended in Laemmli buffer and loaded on SDS-PAGE. Western blot was performed using an anti-WC-1 affinity purified antibody. (**D**) DNA sequence used as cold probes for the EMSA competitive assay (**D**) based on (**A**). The upper sequence is a wt LRE region of the *al-3* promoter. In the lower sequence, the GATA is mutagenized. (**E**) EMSA assay was performed by incubating the labelled double-stranded oligonucleotide with no nuclear extract (lane1), an equal amount of nuclear extract (10 μg) derived from dark growth mycelia (lane 2), or mycelia induced to light for 20 min. (lane 3). The specificity of the binding was verified by co-incubating the labelled oligonucleotide and nuclear extracts obtained from dark growth mycelia or light-treated for 20 min as following. Samples were incubated in the presence of a 10- or 50-fold excess of cold-specific competitor (lanes 4–5, 8–9), or 10- or 50-fold excess of unlabeled specific competitor harboring mutations in both the GATA motifs (lanes 6–7, 10–11).

Many experiments suggest that the WCC recognizes specific promoter-binding sites called Light Responsive Elements (LREs) by interaction with the GATN sequences [5,26,32]. However, the particular role and involvement of the two pairs of imperfect GATN repeats in light-mediated responses are not thoroughly investigated. To verify our working conditions, we first tried to confirm the binding of WCC to the LRE of *al-3*. We chose the *al-3* gene because it is one of the most responsive genes to light. Additionally, it was already proven that WCC could bind to its LREs [5]. We confirmed the importance of WCC in the promoter assembly complex in light-dependent processes by an Electrophoresis Mobility Shift Assay (EMSA). Nuclear extracts of wt *N. crassa*, either grown in constant darkness or subjected to light pulses, were incubated with a radioactive probe corresponding to a sequence containing GATA elements of the *al-3* promoter (Figure 1A). Our results showed that two complexes were present (Figure 1B), a slow migrating complex (upper band shift, SMC lines 6–7) and a fast migrating complex (lower band shift, FMC 6–7), which were DNA-GATA binding proteins (D-GBPs). The SMC was the major complex present in *Neurospora* nuclear extracts and did not show significant differences between dark and light extracts. 

Conversely, the level of the FMC was higher after light pulse (Figure 1B lines 6–7). Our data confirmed the presence of two different complexes (low migrating and fast migrating). Different from what was observed before, we could already detect the slow migrating complex (SMC), in the dark [12,26]. Previous investigations suggested that a preassembled WCC complex could be present in the dark, which might justify our observation [19]. Lack of WC-1, but not WC-2, drastically decreased the major complex binding activity (SMC) and confirmed the pivotal role of this protein (Figure 1B lines 2–5). On the other hand, the FMC was abolished after light treatment in strains deficient for *wc-2*, suggesting that an alternative complex may be present with WC-2 as the core (Figure 1B lines 4–5). Since WC proteins are essential for the stabilization of the two slow and fast D-GBP complexes, we aimed to confirm whether the role of WC-1 in the complex formation was structural. Therefore, we immunoprecipitated WC-2 from the total extract obtained from *Neurospora* wt, *wc-1 null* (negative control), and *wc-1 wmn*, a mutant form of WC-1 previously described [23], which is a shorter version of the protein (around 100 kDa, Figure 1C, Figure S1). WC-1 was specifically coprecipitated with WC-2 only in the wt strain, whereas *wc-1 wmn* was not able to interact with WC-2, confirming that the C-term region of WC-1 was necessary for the formation of the complex (Figure 1C).

Moreover, a super shift of WC-1 was observed after light pulse, suggesting that protein phosphorylation can occur after illumination as already seen (Figure 1C; [19]). To prove the specificity of the results, we performed a competitive EMSA followed by coincubation with either wild-type or mutagenized cold probe, where both GATAs were abolished (Figure 1D). The presence of the two complexes was confirmed in native experimental conditions (Figure 1E lanes 2–3), and it was absent in the negative control (Figure 1E lane 1). 

Excess amounts of cold oligonucleotides corresponding to the wild-type *al-3* competed with the radioactive ones, causing a decreased autoradiographic signal either in the dark or after the light pulse (Figure 1E, lanes 4–5, 8–9). The decrease was dose-dependent and related to the increased amount of cold probe used in the coincubation (10×, 50×). 

Conversely, the mutagenized cold probe did not compete with the radioactive one in the formation of the SMC, although it could still compete for the assembling of the FMC (Figure 1C, 6–7, 10–11). Taken together, these results evidenced that the major D-GBP (SMC) formed a complex specifically with the *al-3* promoter DNA probe both in light and dark conditions. The FMC was reduced with both excess amounts of cold oligonucleotides corresponding to the wild-type *al-3* promoter and with the same oligonucleotide harboring a point mutation in the GATA elements, which suggested that other promoter regions might be involved in the assembling complex. 

### 2.2. Proximal GATA Sequence in Light Responsive Elements (LREs) is Involved in a Repressive Mechanism of Regulation

**Figure 2:**
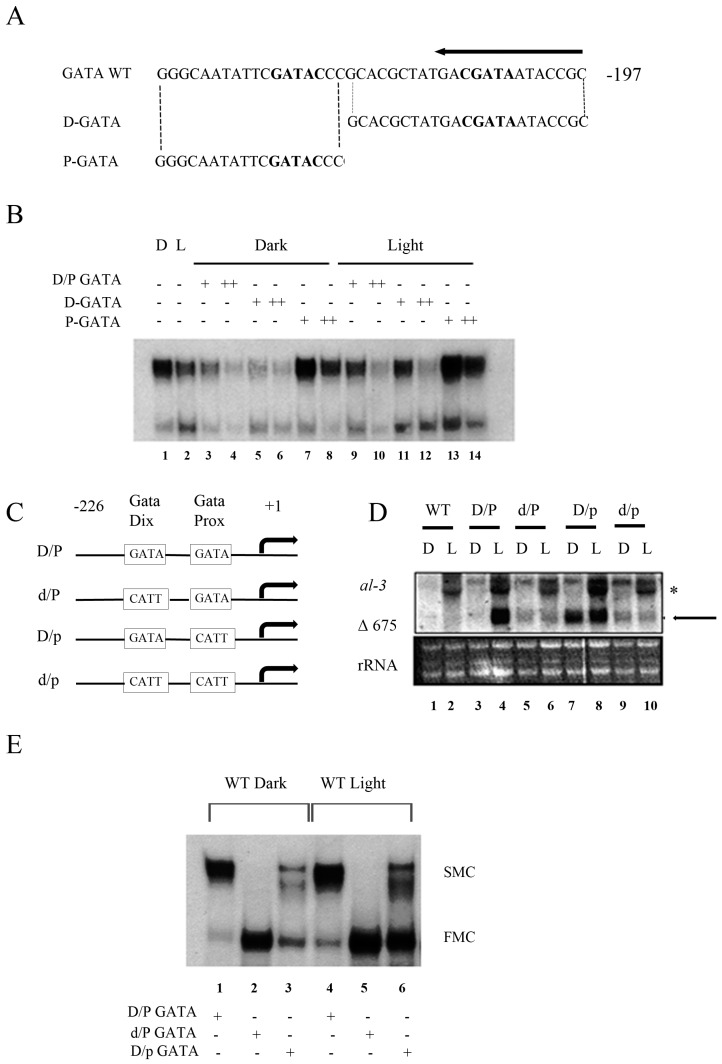
Role of proximal and distal GATA sequences in dark/light shift. (**A**) Schematic representation of *al-3* promoter GATA region that was used for the EMSA. The arrow indicates the direction towards the transcriptional start site. (**B**) EMSA was performed on *N. crassa* nuclear extracts of mycelia grown in the dark or after light pulse and incubated with the *al-3* promoter GATA regions as the probe (lanes 1–2). Competition assay was performed by co-incubating labelled GATA with increasing concentrations (+ = 10-fold, ++ = 50-fold) of each indicated cold competitor sequence (lanes 3–4, 9–10: D/P GATA; lanes 4–5, 11–12: D-GATA; lanes 6–7, 13–14: D-GATA). (**C**) A diagrammatic representation of the exogenous *al-3* promoter bearing mutations of the GATA motifs. (**D**) Representative Northern blot analyses of *Neurospora* transformants containing a shortened version of the *al-3* gene. Asterisk: endogenous *al-3* expression. Arrow: Δ 675. (**E**) EMSA was performed using the nuclear extract of wt *N. crassa* mycelia grown in the dark (lanes 1–3) or light-treated for 20 min (4–6). Competition experiment was performed by adding cold probes as indicated in the figure.

Our next goal was to understand the specific role for both the distal GATA (D-GATA) and proximal GATA (P-GATA), involved in the assembling complex. Therefore, we performed a competitive assay using as a competitor either cold probe carrying D-GATA or P-GATA (Figure 2A), as in Figure 1E. A full-length cold probe containing both GATAs was used as a positive control to show an effective competition with the radioactively labelled one either in the dark or after the light pulse (Figure 2B lanes 3–4, 9–10). Interestingly, the SMC was unaffected by P-GATA oligonucleotide (Figure 2B lanes 7–8, 13–14), whereas D-GATA was able to compete for its binding activity (Figure 2B 5–6, 11–12). These in vitro results together suggested that the D-GATA was more critical for assembling the major nucleoprotein complex, whereas P-GATA was not relevant in this process. In order to understand the in vivo role of each GATA repeat, we transformed wt *Neurospora* strains with a construct containing a shorter version of the *al-3* gene (used as reporter), cloned in frame with its own promoter, containing distal and proximal GATA sequences either wt or subjected to an in vitro site-directed mutagenesis (Figure 2C). The obtained strains were either kept in the dark or illuminated for 20 min, and then RNA was extracted and followed by Northern blot analysis. The endogenous *al-3* RNA was induced in all strains after illumination, confirming that these strains were still able to perceive light normally (Figure 2D, asterisk), whereas the expression of the shorter version of *al-3*, called Δ675 (Figure 2D arrow), was dependent on the GATA that was specifically mutagenized. Wt strain not transformed (negative control) did not show any signal for Δ675 expression, neither in the dark nor after illumination (lanes 1–2). A transformed strain, harboring D-GATA/P-GATA wild-type motifs (positive control), showed Δ675 expression only after light pulse, suggesting that the construct per se was able to respond to the light (lanes 3–4).

In strains harboring either the d-GATA single mutation or d-GATA/p-GATA double mutations (lanes 5–6, 9–10, respectively), Δ675 expression was abolished both in light and in dark conditions. This confirmed that the distal GATA was necessary for a proper assembling of the nucleoprotein complex, which drove light-dependent gene expression. Moreover, because the d-GATA mutant alone resembled the phenotype of the double mutant d/p GATA, D-GATA seemed to be dominant on P-GATA. Surprisingly, the effect of a mutagenized P-GATA alone was entirely different (Figure 2D, lanes 7–8). Constitutive Δ675 expression was observed both in the dark and after a light pulse in a comparable way. This suggested that in the absence of the P-GATA, RNA expression of light-responsive genes became light-independent. So far, it was conveyed the idea that GATA sequences were necessary for recruiting the WCC and starting gene expression. Our data evidenced that the proximal GATA was not required for gene transcription activation; otherwise, when mutagenized, it would have shown the same effect showed by distal GATA mutagenesis in terms of gene expression. Therefore, the most convincing hypothesis is that proximal GATA might be involved in a regulative mechanism that allowed *al-3* gene transcription activation to happen only after a precise light pulse. To investigate further the role of the GATA motives, we performed EMSA experiments. We incubated nuclear extracts obtained from *Neurospora* grown either in the dark or light and treated for 20 min with an oligonucleotide corresponding to either the wild-type GATA repeats or mutagenized D-GATA (d/P) or P-GATA (D/p) (Figure 2E). Oligonucleotides containing the wt GATA motif as a probe were able to make nucleoprotein SMC complexes both in dark and light conditions (Figure 2E lanes 1 and 4), while FMC was assembled mostly after light treatment. The major complex SMC was utterly abolished by using an oligonucleotide with a point mutation in the D-GATA element, both in dark and light conditions (Figure 2E, lanes 2 and 5), confirming again that this motif was required for a functional DNA–protein complex that could be responsive to light. 

Moreover, a substantial accumulation of the FMC was observed. Interestingly, mutation of the P-GATA element resulted in a consistent increase of the FMC in both dark and light conditions compared to the wt probes and, conversely, a concrete decrease of the SMC. These data altogether described two different behaviors of the proximal and the distal GATA sequences. The distal GATA seemed to play a structural role to assemble a major functional complex ready to drive gene transcription in a light-dependent way. Interestingly, the proximal sequence appeared to play a functional role, ensuring that gene transcription mediated by the WCC took place only after the light pulse. Indeed, when P-GATA was mutagenized, *al-3* expression was already promoted in the dark.

### 2.3. Neurospora Strains Lacking the WC-1 Zinc Finger Domain Show Light-Related Phenotypes Already in the Dark 

**Figure 3:**
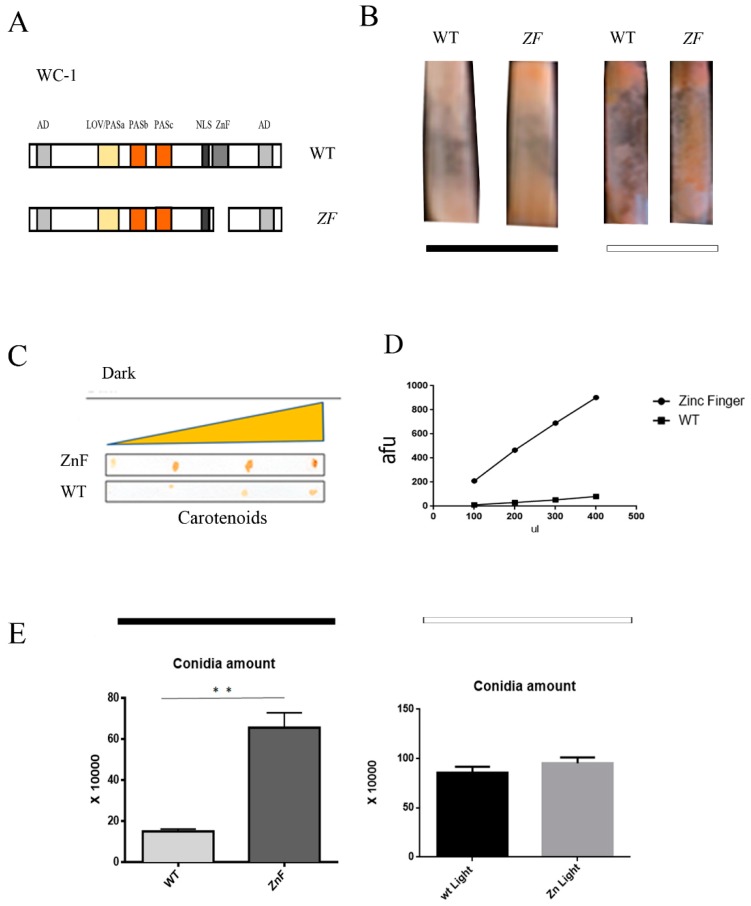
WC-1 zinc finger domain is required for repressing in the dark-/light-dependent phenotypes. (**A**) Schematic representation of functional domains of WC-1 Myc and WC-1 Myc zinc finger deleted proteins. (**B**) Wt WC-1 Myc and WC-1 Myc ZnF ∆ strains were grown in slant for 4 d in the dark, and carotenoid accumulation was checked right after, without giving illumination (left) or after the light pulse (right). (**C**) An increasing amount of conidia resuspended in water (5, 10, 20, and 25 µL) was spotted on a nitrocellulose filter to observe the increment of the orange color corresponding to carotenoid content. (**D**) The carotenoid content was analyzed by densitometry quantification and graphically represented. (**E**) Conidia count in wt and zinc finger deleted strains were measured using a Burker cell counter. The data are shown as the mean ± S.E.M. from three different biological replicas. *t*-test: ** *p*-value < 0.0023.

Our data indicated the P-GATA element might play a functional role in mediating gene activation strictly correlated with a light pulse. To investigate this new dark regulation, we aimed to analyze (*in trans*) if one member of the WCC could be involved in the same pathway. Strikingly, WC-1 seemed to always be recruited in both the dark and light at the promoters of the light-regulated genes, while WC-2 recruitment appeared time-dependent [29,33]. Therefore, we elected WC-1 as the best candidate to function as a repressive factor for the transcription of light-regulated genes. Several WC-1 mutants defective in light perception were isolated [11]. We focused on the WC-1 Myc Zinc Finger domain (here called WC-1 Myc ZnF ∆, Figure 3A). We chose the WC-1 Myc ZnF ∆ mutant because any attempt to show that the WC-1 zinc finger domain worked as a transcriptional activator failed [11,30,31].

We grew *Neurospora* WC-1 Myc (wt littermate control) and WC-1 Myc ZnF ∆ strains for three days in the dark. Subsequently, we photographed them directly after illuminating the strains for 20 min. As expected, the wt strain not subjected to a light pulse showed a pale phenotype (Figure 3B left panel, black bar, wt). Surprisingly, ZnF ∆ showed a peculiar mycelia pink phenotype in the absence of a light pulse in slants where *Neurospora* strains were grown in the total dark (Figure 3B left panel, black bar ZnF ∆; Figure S2 left panel). 

After the light pulse, both strains showed a pink phenotype (Figure 3B right panel, white bar, wt and ZnF ∆; Figure S2 right panel), suggesting that the average dark/light shifting in carotenoid accumulation was active in the wt but not in the ZnF ∆ mutant. Another light-dependent target, the conidiation, was also aberrant in the ZnF ∆ strain. We inoculated wt and ZnF ∆ conidia in a solid medium, and we grew them for eight days. Finally, the amplified conidia not illuminated were filtered in water and collected. We subsequently spotted them on nitrocellulose. Increasing volume for the two strains evidenced a more significant amount of conidia in the ZnF ∆ strain compared to wt (Figure 3C,D). This result was also confirmed by quantification of the conidia number in the Burker cell counter. Our results showed that the ZnF ∆ mutant was characterized by a ten times larger amount of conidia than wt (Figure 3E, left histogram, black bar). However, after a light pulse, the amount was comparable between the two different phenotypes (Figure 3E, right histogram and white bar). Our data indicated that in the absence of the zinc finger domain, the repression of carotenoid expression and conidiation in the dark was abolished. The WC-1 zinc finger domain was not essential for light responses as described before [11]. Moreover, overexpression of WC-1 did not influence light-dependent responses [31]. Therefore, the most convincing conclusion was that the WC-1 zinc finger domain was required as a repressor in dark conditions.

### 2.4. Constitutive Gene Transcription and Chromatin Acetylation in the Dark in Myc WC-1 Zinc Finger Deleted Domain

We observed that mutagenesis of the P-GATA element caused a strong *al-3* RNA expression in the dark (Figure 2D). Similarly, the WC-1 ZnF ∆ mutant was characterized by constitutive carotenoid biosynthesis already in the dark (Figure 3B). It seems P-GATA and zinc finger of WC-1 might play a *cis/trans* role in this repressive mechanism during the dark. To strengthen our hypothesis that WC-1 was directly responsible for the transcriptional repression of light-inducible genes in dark conditions, we further analyzed *al-3* expression in mycelia obtained from WC-1 Myc and WC-1 Myc ZnF ∆ mutants grown in the dark and subsequently subjected to a light pulse of 20 min.

**Figure 4:**
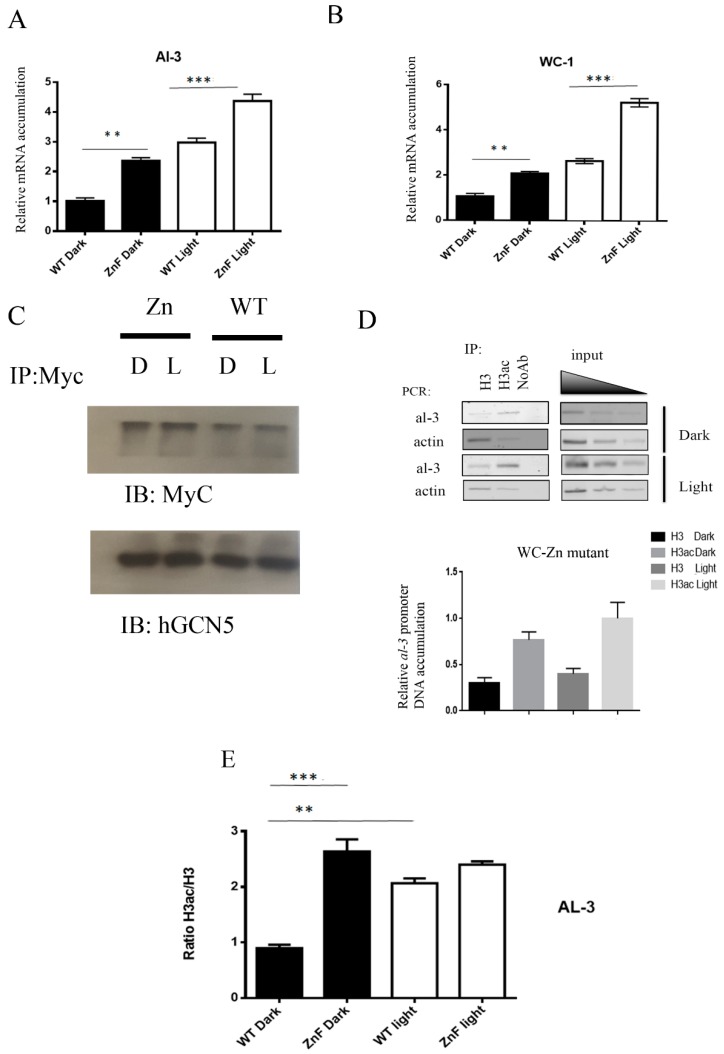
Zinc finger deletion influences increased RNA accumulation and histone acetylation of light-dependent genes in the dark. RNA abundances of *al-3* (**A**) and *wc-1* (**B**) were measured using real-time PCR in the littermate wt, and zinc finger deleted mutant strains, kept either in the dark or after illumination (light 5 min, dark 20 min for a total of 25 min after the light pulse). Data showed a different biological replica that was plotted on a graph with the mean ± S.E.M. from three different experiments. Two-way Anova, Bonferroni: ** *p*-value < 0.05. (**C**) WC-1 was immunoprecipitated using a commercial anti-Myc antibody, and the coresolution of NGF-1 was detected using hGCN5 antibody either in wt WC-1 Myc or WC-1 Myc ZnF ∆ mutant. Immunoprecipitation was performed starting from 400 µg of mycelia grown for 3 d in the dark before harvesting or exposed to saturated light (as in (**A**) and (**B**)). (**D**) A ChIP assay was performed on extract obtained from mycelia grown for 3 d in the dark before harvesting or exposed to saturated light. Anti-H3 and anti-H3-acetylated were used for immunoprecipitating the chromatin. PCR was performed on the free DNA after decrosslinking and further purification. Input: three different dilutions of total input were used for PCR amplification (1/4, 1/8, and 1/16). PCRs were finally quantified by densitometry and plotted on a graph. The data are the mean ± S.E.M. from three independent biological experiments (lower panel). (**E**) H3ac/H3 ratio was measured in wt and zinc finger strains, and data were plotted on a graph to compare accumulation of acetylated form of H3 in both strains in both environmental conditions (dark, light). The plotted data were obtained from three different biological replicas. Two-way Anova, Bonferroni: ** *p*-value < 0.05; *** *p* < 0.001.

Gene expressions of *al-3* and *wc-1* (as control) were analyzed by quantitative PCR. We observed an increased level of both *wc-1* and *al-3* expressions after light pulse in wt strains. Conversely, *al-3* or *wc-1* gene expression was almost three times higher in the ZnF ∆ strain in the dark compared to wt (Figure 4A,B), although *al-3* and *wc-1* transcriptions further increased after light treatment in this strain (Figure 4A,B). This result was consistent with the high level of carotenoid previously observed in the WC-1 ZnF ∆ growth in the dark compared to the littermate wild type. As known, light-dependent gene transcription in *N. crassa* is associated with a change in chromatin acetylation after the light pulse. Besides, it was previously demonstrated that this was related to physical interaction between NGF-1 and WC-1 [22,23]. Therefore, we tested whether WC-1 zinc finger deleted protein was still able to interact with NGF-1. Thus, protein extracts were prepared from both wt WC-1 Myc and WC-1 Myc ZnF ∆ strains collected in the dark or 20 min after light pulse, corresponding to the peak of H3acK and *al-3*/*wc-1* RNA expression. We performed an immunoprecipitation assay, and the heterodimer WC-1:NGF-1 was observed in either the wt or ZnF ∆ mutant without any specific difference between dark and light (Figure 4C). This was in agreement with previously published data, which demonstrated that the WC-1:NFG-1 interaction depended on the LXXLL (L for leucine and X for any other amino acid) motif localized at the C-terminal region of WC-1 placed before the zinc finger domain [23]. 

Since WC-1 was still able to interact with NGF-1, we further focused on light-dependent chromatin acetylation of the *al-3* promoter to understand if there was a link between high mRNA expression in the dark and chromatin remodeling. Chromatin prepared from the ZnF ∆ strain grown in constant darkness or 20 min after the light pulse was immunoprecipitated using anti-acetyl H3 and anti-H3 for normalization, and then DNA was analyzed by PCR using primers recognizing the *al-3* promoter (Figure 4D upper; Figure S3A,B). Consistent accumulation of acetylated histone H3 was observed already in the dark (Figure 4D lower). Results were compared with wt strains (Figure S3B). In the wt strain, the H3 acetylation level at the light-responsive region (LRR) of the *al-3*, measured as a ratio between the acetylated against the total form of H3, increased after the light pulse (Figure 4E). H3 acetylation at the *al-3* promoter in the zinc finger mutant was around three times higher in dark conditions as compared to the wt strain. Additionally, the zinc finger mutant exhibited comparable H3 acetylation of the LRR between dark and light conditions (Figure 4E). Altogether, our data indicated that in dark conditions, the WC-1 zinc finger domain was required for appropriate light-dependent NGF-1 chromatin remodeling, which opened the chromatin structure and allowed the gene expression of *al-3*.

## 3. Discussion

In earlier work, it was found that the GATN-containing proteins WC-1 and WC-2 regulated light-induced gene expression in *Neurospora* by heterodimerization and assembly of the WC Complex (WCC) [12,25,26,27,28].

WCC is recruited onto the promoter of light-responsive genes via interaction with LREs [10,12,25,26]. LREs are made by two imperfect GATN (N indicates any other nucleotide) repeats [34]. It was identified in the promoter of the *albino-3* gene (a light-dependent gene involved in carotenoid biosynthesis) a pair of GATA repeats which are responsive to the light [35]. The distal GATA (D-GATA), also known as C-box, is also included in regulation of the circadian clock in *Neurospora* [12]. Less known is the role of the proximal GATA (P-GATA) in terms of light responses. We performed an EMSA assay to observe DNA–protein complexes between LREs and WCC. Our EMSA provided evidence for the presence of two principal D-GBP complexes differently distributed in dark and light conditions: a slow migrating complex (SMC) and fast migrating complex (FMC) (Figure 1B). These data are partially in agreement with previous observations, where two complexes were shown as well after EMSA [12,26]. Our view suggested that the major complex was present both in light and dark conditions, whereas the lower complex was mostly stimulated after the light pulse. The major complex disappeared in *wc-1* ko strains suggesting a structural role for this protein. Indeed, when WC-1 is not present or it lacks its C-terminal, WCC is not assembled, and gene transcription of light-dependent targets is abolished (Figure 1C; [11,23]). 

Lack of WC-2 is responsible for loss of the minor complex FMC after the light pulse (Figure 1B). Since WC-1 is constantly recruited on the chromatin of its target genes, while WC-2 recruitment is partially time-dependent, our results suggest that another light-dependent complex might be assembled, where WC-2 is the core that supports the major complex made by the known WCC. Moreover, our data indicate that only D-GATA seems to play a structural role. Indeed, in a competitive EMSA, cold D-GATA, but not cold P-GATA, was able to compete in the formation of the radioactive nucleoprotein complex when a labelled sequence containing both GATA repeats was used for the assay (Figure 2B). 

Moreover, EMSA performed with probes, where both GATA were singularly mutagenized, highlighted that the D-GATA was more prone to interact with SMC, while the P-GATA seemed to interact with the FMC (Figure 2E). Our data were partially in disagreement with those by He and Liu, which showed that the SMC appeared after light pulse, whereas the FMC disappeared. In any case, control experiments we performed on *wc-1* and *wc-2* mutants supported our results. Indeed, the SMC interacts with the D-GATA, and it is functional in the presence of WC-1. Published data by both Dunlap and Liu evidence how WC-1 is fundamental in total darkness for the circadian clock, and the D-GATA plays a pivotal role in recruiting clock core components. 

Moreover, D-GATA is also a consensus sequence belonging to the LREs, indeed, when it is mutagenized, our data show that light responses are abolished. From this point of view, it is logical that a complex driven by WC-1, which interacts with D-GATA, should be observable both in the dark and in light because it would drive the transition from the circadian cycle to light resetting. Since FMC assembling is light-dependent and WC-2 drives it, and WC-2 recruitment is not constant as already shown in the circadian cycle [29,33], it is logical to suppose that this complex accumulates more after illumination. In any case, to understand the discrepancy with older published data, more experiments need to be carried out. Since P-GATA interacts with the minor complex FMC, which is light-regulated, it could mean that P-GATA could play a key role in the dark/light shift. Indeed, when we transformed *Neurospora* strains with vectors carrying different forms of the GATA repeats (either wt or both or singularly mutagenized) and monitored light-dependent gene expression using a short form of *al-3* mRNA (Δ675) cloned in frame with the GATAs as a reporter, we observed something unexpected. 

Mutagenesis of D-GATA abolished any light responses (as expected), while mutagenesis of P-GATA constitutively activated Δ675 gene expression already in the dark (Figure 2D). Altogether, our results suggested that D-GATA might be involved in a structural complex present in both dark and light, which might suit the idea that it is also essential in the regulation of the circadian clock mediated by the WCC in constant darkness. On the other hand, P-GATA might be part of a functional complex that favors gene transcription only after the light pulse. The idea of a repressive mechanism involved in light responses was partially assumed before [11]. Here, we were able to give more insight into that. 

We focused our attention on WC-1 because our data showed that this protein had a structural role in assembling the complex. Moreover, while WC-2 was a clear transcriptional activator, any attempt to prove that WC-1 was also a transcriptional activator failed [11,30,31]. Our data suggest that the WC-1 zinc finger domain is not responsible for any transcriptional activation, but it might be involved in the formation of a repressive transcriptional complex. Indeed, any light-dependent response is decoupled by the signal in these mutant strains. For instance, conidiation and carotenoid biogenesis are already high in the dark phase (Figure 3). Carotenoids biosynthesis is one of the most common early responses [10], and our data suggest that at least *al-3* gene expression is aberrantly high already in the dark (Figure 4A). The WC-1 ZnF ∆ mutant protein is still able to interact with NGF-1 (Figure 4C), which is the histone acetyltransferase responsible for acetylating histone H3 in order to mediate chromatin opening and further gene transcription of light-regulated genes [23]. What we found interesting was that the H3 acetylation ratio at the *al-3* promoter was almost three times higher in the ZnF ∆ mutant when compared to wild type in dark conditions (Figure 4E), which justified that gene expression was already taking place without a light pulse. Altogether, these pieces of evidence suggest that WC-1, through its zinc finger domain, plays a repressive role in regulating light-dependent responses.

How might an active repressor function of WC-1 block transcription? 

There is some evidence that shows that the absence of RCO-1/RCM-1 prevents gene transcription in the dark, and lack of a functional RCO-1 or RCM-1 leads to gene derepression in the dark [36]. The *rco-1* and *rcm-1* mutants reflect the WC-1 ZnF ∆ phenotype. Besides, the authors suggested a functional competition for the promoter binding sites (i.e., at *al-3*) between RCO-1-/RCM-1-containing repressor complexes (which prevent transcription in the dark) and other unknown activating chromatin remodelers. In previous works, it was demonstrated that the chromatin remodeler was NGF-1 [22,23]. Additionally, it was described that activation of WC-1 in the dark was promoted by Protein Kinase C (PKC)-mediated zinc finger phosphorylation [37], which would mean that some rearrangement of WCC might happen after this phosphorylation. Our data, together with those related to cited papers, suggests that in dark conditions WC-1 might lead to the formation of a basal transcription repressor complex recruited onto the distal GATA consensus of light-responsive promoters that might prevent gene transcription in the absence of light. Indeed, when the D-GATA is disrupted, there is no complex driven by WC-1 neither in the dark nor after the light pulse. Somehow, this repressive complex assembled on the D-GATA might recognize and mask the P-GATA and prevent its exposition to the light-driven complex, with WC-2 as the core, in the absence of a light pulse. Indeed, when P-GATA is mutagenized, transcription of light-inducible genes is active in the absence of light pulse, which means that a transcriptional start site is available to the RNA polymerase. This observation might suggest that a repressive complex formed by WC-1, RCO-1, or RCM-1 could be recruited onto the P-GATA, and it might create a steric hindrance to avoid relocation of WC-2 and polymerase recruitment before the light pulse. Indeed, the same phenomenon is observed in WC-1 ZnF ∆, *rco-1*, and *rcm-1* suggesting that the *cis*-element (P-GATA) and *trans*-acting factors (WC-1, RCO-1, and RCM-1) are components of the same molecular pathway.

**Figure 5:**
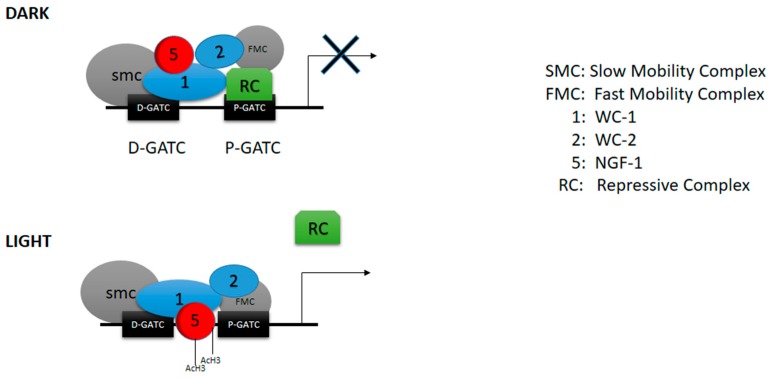
Proposed molecular model. Schematic representation that summarizes the data discussed in the paper. White collar complex (WCC), slow mobility complex, and fast mobility complex form a preassembled molecular scaffold, present already in the dark. Moreover, the WCC binds the acetyltransferase NGF-1. The presence of a putative repressive complex is suggested even if it is not clear what proteins are involved. After light pulse, the WC-2:FMC complex binds the promoter, and the repression is abolished. The acetyl transferase NGF-1 is placed onto the promoter in order to modify the light-dependent chromatin acetylation, and gene expression is promoted.

On the other hand, we tried to understand how WC-1 repressive functions could influence the dark–light transition. Our data provide evidence that WC-1 interacts with NGF-1 with or without the zinc finger. Although chromatin acetylation is promoted only after light pulse in wt strain, chromatin hyper-acetylation is already observed in the dark in the mutant lacking the zinc finger domain, and, consequently, gene transcription is turned on without any light pulse. These results suggest that even if WC-1 needs a functional LXXLL for interacting with NGF-1, only a functional zinc finger promotes a proper dark–light transition through NGF-1 relocation on the chromatin right after the light pulse, even if it is not clear how. This model (Figure 5), still speculative in some aspects, is consistent with our findings, and it explains the results presented here. This hypothesis is also compatible with present-day literature, and it may become a framework for future experiments.

## 4. Materials and Methods 

### 4.1. Neurospora Strains and Growth Conditions

#### 4.1.1. Neurospora Strains Used in the Paper

-Wild-type strain 74OR23-1A (FGSC 987): Fungal Genetic Stock Center (Kansas City, KS). 

-*wc-1* KO mutant (matA; his-3; bd; wc-1null; FGSC 3081): J. Dunlap (Dartmouth Medical School, Hanover, NH).

-WC-1 Myc strain: obtained by transformation of *wc-1* KO with pDE3dBH vector carrying the entire wc-1 Myc tagged under the qa2 promoter [38]. 

-WC-1 Myc ZnF ∆: the strain expressed a WC-1 protein missing the ZnF ∆F (Yi Liu, PhD. UT Southwestern Medical Center. Dallas, TX, USA) [11]. 

#### 4.1.2. Medium and Growth

-Liquid growth: Vogel’s minimal medium supplemented with 1.5% sucrose (Sigma, St. Louis, MO, USA) was used for amplifying *Neurospora* mycelia starting from inoculation of 1 × 10^6^ conidia. Cultures were incubated for 48–72 h in the dark at 28 °C. 

-Solid growth: same conditions used for liquid growth with the addition of Agar 14 g/L (Difco)

#### 4.1.3. Preparation of Dark-Grown Samples and Light Stimulation

Mycelia were collected by filtration under a red safety lamp and frozen in liquid nitrogen. Light stimuli were performed by illuminating mycelia for 5 min with saturating light (10 W/cm^2^) followed by incubation in the dark for different time intervals before collection under a red safety lamp. 

The time intervals indicated in the figures refer to the time elapsed after exposure to the light pulse stimulus.

### 4.2. Conidia Count and Carotenoid Content

*Neurospora* strains were grown in 250 mL flasks containing 100 mL of solid Vogel’s minimal medium supplemented with 1.5% sucrose and then recovered in 25 mL of water. A dilution of conidia was observed with a Burker cell counter, and the count was performed according to the manufacturer’s formula:

$$\left[\frac{N.\mathrm{conida}}{4}\right]*16*10^{4}*\mathrm{dilution}\;\mathrm{factior}.$$

The chromatic effects (qualitative data) of the dilution were observed by spotting the conidia on a nitrocellulose filter.

### 4.3. Immunoprecipitation Assay

Experiments were performed as in [24]. Total protein samples from frozen mycelia were extracted with lysis buffer A (50 mM 4-(2-hydroxyethyl)-1-piperazine ethansulfonic acid (HEPES), pH 7.4, 137 mM KCl, 10% glycerol containing 1 mM phenyl-methyl-sulfonyl-fluoride (PMSF), 1 mM ethylen diamine tetraacetic acid (EDTA, Sigma) and 1× protease inhibitor cocktail tablets (Roche, Indianapolis, IN)). Protein extracts (400–600 μg) were precleaned in advance with protein A Sepharose (Roche) for 1 h at 4 °C to minimise nonspecific interactions. The precleaned extract was incubated over night at +4 °C with 5 μL of affinity-purified anti-Myc antibody (Santa Cruz Biotechnology, Santa Cruz, CA) followed by coincubation with 10 μL of protein A-Sepharose for 3 h at +4 °C. After incubation, the beads were washed five times with 500 μL of lysis buffer containing 0.05% Triton X-100. The beads were then resuspended in Laemmli’s sample buffer, boiled for 10 min at 95 °C, and loaded on 10% SDS–PAGE gel.

### 4.4. Chromatin Immunoprecipitation Assay (ChIP)

Chromatin immunoprecipitation assay (ChIP) experiments were performed as described in [23]. Crosslinked *Neurospora* samples were immunoprecipitated with either antibody against H3 (06-755; Upstate Biotechnology, Charlottesville, VA) or anti-acetyl H3 (06-599; Upstate Biotechnology) and anti-hGCN5 (Santa Cruz Biotechnology). The product was decrosslinked, and DNA was extracted. The obtained template was subjected to PCR using the following primers:
Promoter region of *al-3*(5′-AGA TAG ATC TCT TGG CCT TG-3′) FW
(5′-CGA TTA TTG GAA ACC CGT CGG TA-3′) RWPromoter region of *actin*(5′-CCT CTC TCA GCC AAA GCA TC-3′) FW
(5′-GAA AGC TTA CCC CAT TGT CG-3′) RW

Actin was used for normalization. Band intensities were quantified by optical density analyses with OptiQuant Software (PerkinElmer Life and Analytical Sciences, Boston, MA). As negative controls, mock precipitations were performed in the absence of antibody. Quantification was performed as follows: ((*al-3*/act) IP/(*al-3*/act) input). Three ChIP replicates were performed on different preparations for each experiment.

### 4.5. Nuclei Isolation

The mycelial pads (2–3 g, wet weight) were ground in liquid nitrogen with a mortar and pestle and suspend in ice-cold buffer A 1× at a ratio of 0.5 mL of buffer for 0.1 g of tissue (5×: 50 mM NaCl, 50 mM Mes pH 6.0, 0.5 mM EDTA, 0.75 mM spermine, 2.5 mM spermidine, 100 mM mercaptoethanol, 1.25 M sucrose, 3% Triton X-100 containing 1 mM phenyl methyl sulfonyl fluride (PMFS) 1 mM EDTA, 1 mM leupeptin, and 1 mM pepstatin A). The crude extract was filtered through cheese cloth and centrifuged at 5000 revs/min for 10 min in an SS-34 Sorvall rotor to pellet the nuclei. The resulting supernatants were removed, and the nuclear pellet was layered onto 15 mL step gradients (Buffer B: 6ml 5× buffer A, 45 mL Percoll) and centrifuged at 7000 revs/min for 5 min in an SS-34 Sorvall rotor to remove cell debris. The nuclear pellet was transferred to a flask and washed with buffer A 1×. The resulting crude nuclear pellets were resuspended in storage buffer (20 mM HEPES, pH 7.4, 25% (*v*/*v*) glycerol, 50 mM NaCl, 1.5 mM MgCl_2_, 0.1 mM EDTA, 1 mM PMSF, 1 mM leupeptin, and 1 mM pepstatin A) and sonicated with three pulses of 10 s each, centrifuged at 13,000 rpm in centrifuge tubes to remove nuclear debris, and samples were stored at −70 °C.

### 4.6. Northern Blot Analysis

Total RNA was extracted from *Neurospora* samples according to the protocol used in Brenna et al. [23]. Mycelia was grown in the darkness for two days and subsequently subjected to appropriate light pulse or collected under dim light. RNA was extracted, and the quality was checked by electrophoresis gel run. Subsequently, the RNA was transferred to Hybond-N membrane (Amersham), hybridized in the presence of 50% formamide after addition of 1.5 × 10^6^ counts per minute (c.p.m.)/mL of Albino-1 ^32^P-labelled probes, and a Northern blot was performed per the manufacturer’s protocol.

### 4.7. Gel Mobility Shift Assay

A double-stranded probe was generated by incubating annealed oligonucleotides (as following):

-CGCCATAATAGCAGTATCGC a

-GCGGTATTATCGTCATAGCGTGCGGGTATCGAATATTGCCC

in the presence of Klenow, (^32^P) dATP, dTTP, dCTP, and dGTP by following standard protocols (Boehringer). Binding reactions for gel shift assays were performed at 28 °C in 20 µL final volume of binding buffer (20 mM Hepes pH 7.4, 50 mM NaCl, 1.5 mM MgCl_2_, and 0.2 mM EDTA) containing 1 µg of bovine serum albumin, 1 µg of poly (dI-dC), 0.5 ng of (^32^P) dATP-labelled double-stranded oligonucleotide probe, and 10 µg of nuclear extract. After 30 min at 28 °C, the reaction mixture was loaded onto a 5% nondenaturing polyacrylamide gel in 0.5× Tris-borate EDTA and subjected to electrophoresis at 12 V/cm at 4 °C. The gel was dried and subjected to autoradiography. The following oligonucleotides were used as a probe or as a competitor in gel shift experiments:
*al-3* promoter, 41 bpGCGGTATTATCGTCATAGCGTGCGGGTATCGAATATTGCCC20 bp GATA distalGCGGTATTATCGTCATAGCG21 bp GATA proximalTGCGGGTATCGAATATTGCCCGATA distal mutatedGCGGTATGAGAGTCATAGCGTGCGGGTATCGAATATTGCCCGATA proximal mutatedGCGGTATTATCGTCATAGCGTGCGGGGAGAGAATATTGCCCGATA mutatedGCGGTATGAGAGTCATAGCGTGCGGGGAGAGAATATTGCCC

Note in the text, in accordance with previous papers, the complementary sequence was shown.

### 4.8. Real-Time qPCR

Real-time qPCR was performed as previously described [39]. Primers used for the cDNA amplification were the following:

Albino-3
AL3 F5’-CTGTTCCGCTTGGGAATCAA-3’AL3 R5’-CGATATGGAAGATAAGTCCGAT-3’

White collar-1
WC-1 F5’-TTGAAAGCCACGGAACATTGT-3’WC-1 R5’-CTGTGTAGAGCAAAAACGGGG

### 4.9. Statistical Analysis

Statistical analysis was performed using GraphPad Prism6 software. Depending on the type of data, either an unpaired t-test or a two-way Anova with a Bonferroni post hoc test was performed. Values were considered significantly different at a *p* value < 0.05.

## 5. Conclusions

In summary, two main complexes regulate light responses processes in *Neurospora crassa*. A major complex with WC-1 as core, called Slow Migrating Complex or SMC, acts as repressor in order to avoid that light regulated genes might be activated independently from the signal. A minor complex with WC-2 as core, called Fast Migrating Complex or FMC, is involved in the light dependent gene activation. While the Distal GATA (D-GATA) consensus in promoter sequences of genes as *albino-3* is involved in the light dependent gene activation, we found that the Proximal GATA (P-GATA) is involved in the repressive regulating mechanism. Finally, we demonstrated that WC-1 acts as repressor through its zinc finger domain. Indeed, when that domain is deleted, phenotypes that we usually observe after light pulse, such as conidiation and carotenogenesis, are already observed in the dark. These phenotypes are a consequence of hyperacetylated chromatin of histone H3, which is a common marker for transcriptional activation. Hyperacetylated chromatin finally promotes expression of genes such as *al-3* already in the dark, without any light stimulus.

## Supplementary Materials

Supplementary materials can be found at https://www.mdpi.com/1422-0067/20/12/2854/s1.
