# Supplementary material for: WC-1 and the Proximal GATA Sequence Mediate a Cis-/Trans-Acting Repressive Regulation of Light-Dependent Gene Transcription in the Dark
Source: Int J Mol Sci. 2019 Jun 12;20(12):2854. doi: 10.3390/ijms20122854 (PMC6628569; doi:10.3390/ijms20122854)
Supplement: Supplementary file 1 [file ijms-20-02854-s001.pdf]

## Supplementar Figures

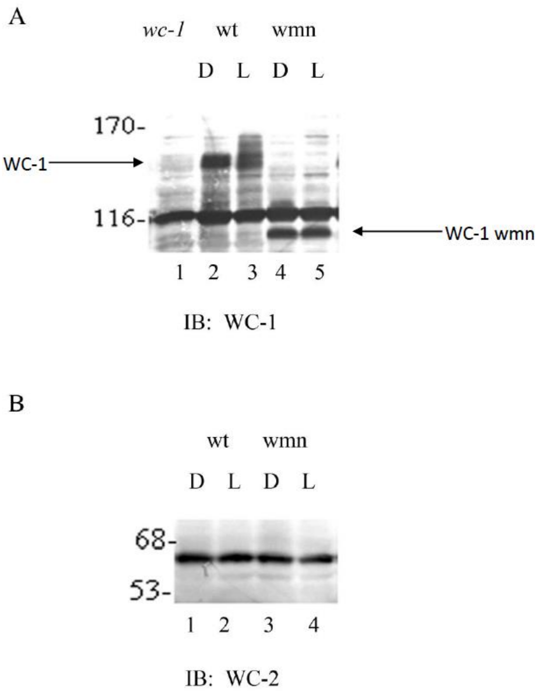


**Figure S1.** (**A**) WC-1 was detected on extracts (input used for IP in Figure 1C) obtained from *wc-1* null mutant (negative control), wt and *wmn* mutant in the dark and after illumination. Arrows indicate different sizes for WC-1 in Wt and *wmn* mutant. (**B**) WC-2 was detected in wt and *wmn* samples.

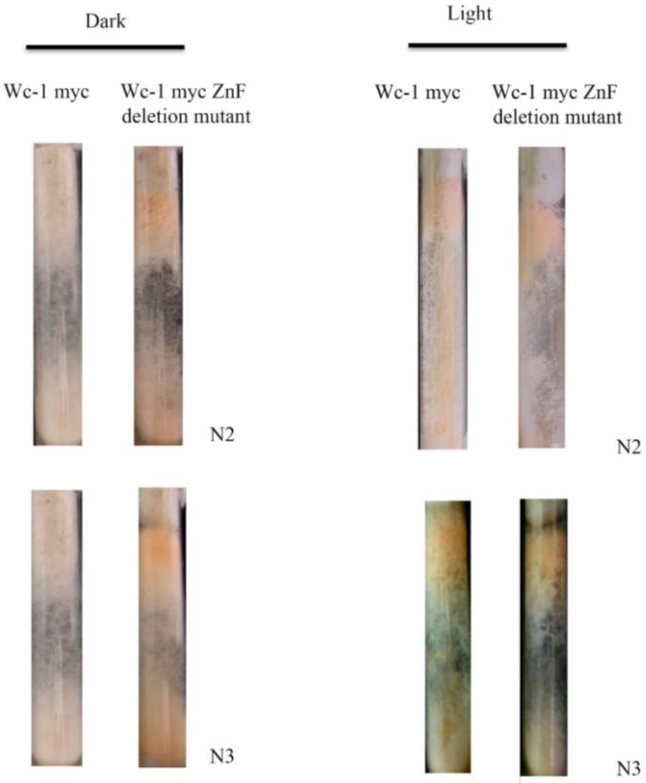


**Figure S2.** Phenotype analysis related to Figure 3B. Replica experiments performed on WT WC-1 Myc and WC-1 Myc ZnF Δ mutant following the protocol described in Figure 3B.

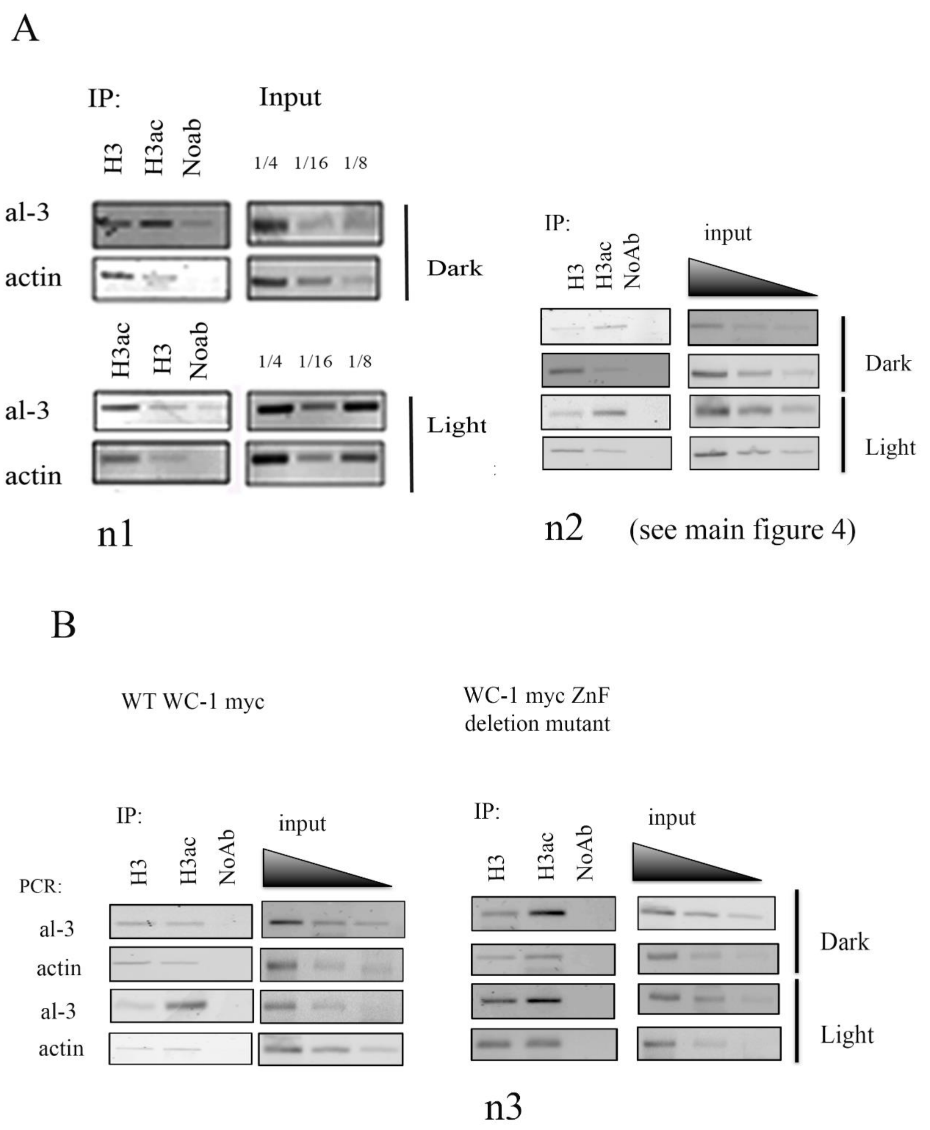


**Figure S3.** (**A**) Row data representing ChIP replicas used for quantification in Figure 4D (n1, n2 and n3 showed in (**B**). Replica n2 is shown in the main figure as representative example. (**B**) Comparison between representative ChIP analyses performed in wt WC-1 Myc and ZnF Δ mutant.
